# Does gene flow aggravate or alleviate maladaptation to environmental stress in small populations?

**DOI:** 10.1111/eva.12768

**Published:** 2019-02-04

**Authors:** Sarah W. Fitzpatrick, Brendan N. Reid

**Affiliations:** ^1^ W.K. Kellogg Biological Station, Department of Integrative Biology Michigan State University Hickory Corners Michigan

**Keywords:** gene flow, genetic drift, *Poecilia reticulata*, RADseq, stress response, thermal tolerance

## Abstract

Environmental change can expose populations to unfamiliar stressors, and maladaptive responses to those stressors may result in population declines or extirpation. Although gene flow is classically viewed as a cause of maladaptation, small and isolated populations experiencing high levels of drift and little gene flow may be constrained in their evolutionary response to environmental change. We provide a case study using the model Trinidadian guppy system that illustrates the importance of considering gene flow and genetic drift when predicting (mal)adaptive response to acute stress. We compared population genomic patterns and acute stress responses of inbred guppy populations from headwater streams either with or without a recent history of gene flow from a more diverse mainstem population. Compared to “no‐gene flow” analogues, we found that populations with recent gene flow showed higher genomic variation and increased stress tolerance—but only when exposed to a stress familiar to the mainstem population (heat shock). All headwater populations showed similar responses to a familiar stress in headwater environments (starvation) regardless of gene flow history, whereas exposure to an entirely unfamiliar stress (copper sulfate) showed population‐level variation unrelated to environment or recent evolutionary history. Our results suggest that (mal)adaptive responses to acutely stressful environments are determined in part by recent evolutionary history and in part by previous exposure. In some cases, gene flow may provide the variation needed to persist, and eventually adapt, in the face of novel stress.

## 1 INTRODUCTION

Population persistence in the face of human‐induced global change often requires tolerance to abrupt environmental stress. What is “stressful” depends largely on the environmental conditions to which a population has adapted. For example, high temperatures and low water availability in desert conditions would most likely kill organisms restricted to cool and wet places. However, natural populations are increasingly exposed to novel stressors (e.g., predators, pathogens, or environmental toxins) or more severe environmental stress than what they have previously faced (e.g., thermal extremes; Hoffmann & Sgro, 2011). Populations that are unable to tolerate novel or increased stress will likely show sharp fitness declines and are vulnerable to extirpation (Fagan & Holmes, 2006; Frankham, 2005; Hoffmann & Hercus, 2000). Identifying the determinants of population persistence, performance, and adaptive potential under increasingly stressful conditions is urgent, given the rapid increase in worldwide population extinctions (Ceballos, Ehrlich, & Dirzo, 2017; Urban, 2015).

Genetic drift in small and isolated populations can increase genetic load due to an increased frequency of deleterious alleles (Charlesworth & Charlesworth, 1987). The expectation that inbreeding depression will be more pronounced in stressful environments compared to benign conditions is generally supported (Armbruster & Reed, 2005; Fox & Reed, 2011; Nowak et al., 2007), though inbred populations may respond to stress differently depending on taxonomic lineage, history of inbreeding, and the type, duration, novelty, and severity of stress (Keller & Waller, 2002; Sandner & Matthies, 2016). If effects of inbreeding depression are exacerbated under stress, the impacts of stress should be generally worse in populations experiencing strong genetic drift. Despite ample literature testing heterozygosity–fitness correlations (Chapman, Nakagawa, Coltman, Slate, & Sheldon, 2009; Szulkin, Bierne, & David, 2010), the relationship between neutral population genetic diversity and stress response, specifically, is equivocal (Hoffmann & Daborn, 2007). However, it tends to be the case that individuals with increased heterozygosity demonstrate a broader range of physiological tolerance and function relative to homozygous individuals (Chapman et al., 2009; Danzmann, Ferguson, & Allendorf, 1988; Forcada & Hoffman, 2014; Samollow & Soulé, 1983). Given increasingly small and isolated populations caused by habitat loss and fragmentation, there is a necessity to further understand generalizability of inbreeding–environment relationships as well as how populations are constrained by the loss of genetic variation (Pauls, Nowak, Bálint, & Pfenninger, 2013).

**Figure 1:**
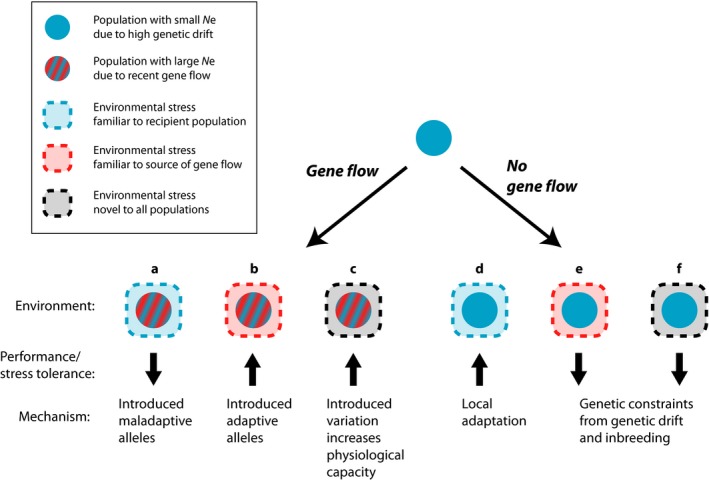
Predicted effects of stress on a small population (solid blue) with low genetic variation (small *N*_e_) depending on recent gene flow history. Populations with gene flow (striped) have increased genetic variation (large *N*_e_). Gene flow populations exposed to an environmental stress familiar to the recipient population such as survival of headwater populations to low‐resource levels (dashed blue square; a vs. d) should be maladapted relative to no‐gene flow populations due to the introduction of maladaptive alleles. Gene flow populations exposed to an environmental stress familiar to the source of gene flow such as higher water temperatures (dashed red square; b vs. e) should be adapted relative to no‐gene flow populations due to the introduction of adaptive alleles. Gene flow populations exposed to a novel environmental stress such as a heavy metal toxin (dashed black square; c vs. f) should be adapted relative to no‐gene flow populations due to lack of genetic constraints imposed by genetic drift and inbreeding in small populations

Gene flow is often considered a source of maladaptation because it can limit genetic and phenotypic differentiation and reduce mean fitness in a population that receives immigration of locally maladapted alleles (Garcia‐Ramos & Kirkpatrick, 1997; Hendry, Day, & Taylor, 2001; Lenormand, 2002). However, the fitness effects of gene flow depend on demography of the recipient population and stability of the environment (Holt & Gomulkiewicz, 1997; Sexton, Hangartner, & Hoffmann, 2014), as well as the shape and steepness of environmental gradients (Bridle, Kawata, & Butlin, 2019). The same level of gene flow that homogenizes and limits adaptation under one set of conditions (i.e., migration–selection balance between medium to large populations in stable environments; King & Lawson, 1995; Hendry & Taylor, 2004; Nosil & Crespi, 2004) may promote adaptive divergence under a different set of conditions (i.e., evolutionary rescue in stressful or rapidly changing environments; Willi, Kleunen, Dietrich, & Fischer, 2007; Aitken & Whitlock, 2013; Apgar, Pearse, & Palkovacs, 2017). Increasing empirical evidence suggests that recently fragmented populations with reduced population sizes stand to receive a demographic benefit from gene flow beyond the addition of immigrant individuals through genetic rescue (Frankham, 2015; Hufbauer et al., 2015; Whiteley, Fitzpatrick, Funk, & Tallmon, 2015). However, the extent to which new gene flow determines capacity to withstand and eventually adapt to stressful environments in the wild has not been explored. If new gene flow provides a means of recovery from inbreeding depression, these populations may show an increased capacity to handle stressful conditions compared to no‐gene flow analogues (i.e., Figure 1b,c vs. Figure 1e,f). On the other hand, if immigration comes from an environment that has not previously encountered a stress that is common in the recipient environment, homogenizing gene flow may constrain locally adaptive stress response (Figure 1a vs. Figure 1d). Here, we provide a case study using Trinidadian guppies, highlighting that gene flow may counteract maladaptive responses to abrupt stress, especially when populations are otherwise constrained by a lack of genetic variation.

Trinidadian guppies are a model system in evolutionary ecology (Magurran, 2005), used for studies on behavior (Hughes, Houde, Price, & Rodd, 2013), rapid adaptation (Reznick, Bryga, & Endler, 1990), and eco‐evolutionary feedbacks (Travis et al., 2014). Guppies have also been used to study organismal stress responses to predation (Fischer, Harris, Hofmann, & Hoke, 2014), chemical toxicity (Park & Heo, 2009), temperature (Muñoz, Breckels, & Neff, 2012), and hypoxia (Poulin, Wolf, & Kramer, 1987), as well as for testing phenotypic and fitness effects of inbreeding (e.g., Sheridan & Pomiankowski, 1997; van Oosterhout et al., 2003). The only study we know of that explicitly tests inbreeding–stress relationships in guppies examined pathogen resistance, documenting higher parasite intensity and less ability to clear the infection in inbred individuals compared to outbred fish (Smallbone, Oosterhout, & Cable, 2016).

Wild guppy populations found in headwater streams in Trinidad are known to harbor low levels of genetic variation due to founder effects and subsequent isolation by waterfall barriers (Crispo, Bentzen, Reznick, Kinnison, & Hendry, 2005; Suk & Neff, 2009; Willing et al., 2010). Waterfalls also limit upstream dispersal of most predatory fish species, resulting in low levels of predation mortality in these environments (Reznick, Butler, Rodd, & Ross, 1996). Guppy populations in headwater streams are characterized by higher densities (Reznick & Bryant, 2007; Reznick, Butler, & Rodd, 2001) and resource limitation (Grether, Millie, Bryant, & Reznick, 2001), and as a result are better adapted to high population densities than guppies from resource‐rich mainstem environments (Bassar et al., 2010; Reznick, Bassar, Travis, & Helen Rodd, 2012). This study leveraged a set of previous translocation experiments that occurred in 2009 (Travis et al., 2014), which initiated gene flow from differentiated guppy populations into two recipient headwater populations with previously low levels of genetic variation. New gene flow caused substantial increases in population growth owing to high hybrid fitness, but mostly did not cause the loss of locally adapted traits (Fitzpatrick et al., 2016, 2017; Fitzpatrick, Gerberich, Kronenberger, Angeloni, & Funk, 2015).

Here, we tested acute stress responses and characterized population genomic differences among small populations found in similar headwater environments with different recent evolutionary histories (i.e., recent gene flow vs. genetic drift). Previous work documented increased genetic variation following gene flow from the upstream translocations using microsatellite data (Fitzpatrick et al., 2016). However, for the research described here, we were especially interested in comparisons between the populations that experienced recent gene flow versus no‐gene flow analogues. Specifically, in this study, we compared stress responses among gene flow recipient populations and the gene flow source population from Fitzpatrick et al. (2016) as well as several headwater guppy populations without a recent history of gene flow that had not been characterized in the previous study. We tested for population‐level differences in stress response to three acute stressors: (a) heat shock; (b) starvation; and (c) exposure to a novel metal toxin (copper sulfate). This set of stressors represented a range of conceivable environmental perturbations that could be relatively easily administered and offered quantifiable responses.

We hypothesized that stress response would depend on recent evolutionary history (gene flow vs. no gene flow) and whether populations had previous exposure to the stress (Figure 1). High temperatures should be novel to headwater populations that occupy cooler waters. However, the gene flow source population originated from a warmer downstream environment, so we predicted populations with a recent history of gene flow would show higher thermal tolerances, possibly due to the introduction of heat‐tolerant alleles (Figure 1b) or due to an overall increase in genetic variation (Figure 1c). We predicted previous exposure to low‐resource environments would lead to higher starvation tolerance in headwater populations (Figure 1d), though gene flow populations may show reduced tolerance due to genomic homogenization from a population adapted to a high resource environment (Figure 1a). Finally, we predicted gene flow populations would show highest tolerance to the metal toxin due to broadly increased physiological capacity caused by an increase in genomic variation and reduction in inbreeding (Figure 1c).

We also generated genomic data from all populations to characterize genetic factors that we would expect to influence responses to stress. We used this genomic data to characterize (a) population genomic diversity and differentiation, with the expectation that diversity would be lower and differentiation from the source population would be higher in headwater populations without a recent history of gene flow; (b) individual inbreeding and number and lengths of long runs of homozygosity, with the expectation that populations without a recent history of gene flow would show more evidence of genome‐wide inbreeding; and (c) the effects of local selective pressures in headwater populations, with the expectation that local selective pressures may maintain regions of genomic divergence (detectable as *F*_ST_ outliers) that could preserve adaptations to headwater‐specific stressors, potentially even in the face of gene flow.

## 2 METHODS

### 2.1 Site descriptions and sampling

**Figure 2:**
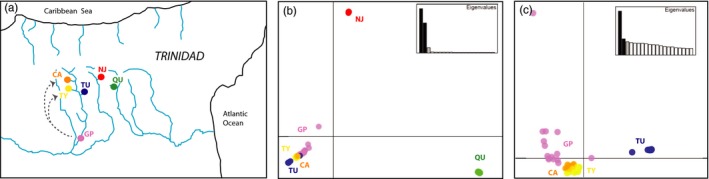
(a) Map showing five focal headwater population sites in Trinidad (CA, NJ, QU, TU, and TY). Gray arrows indicate two sites that received gene flow from a mainstem source site (GP). (b) PCA plot showing overall population differentiation of all populations based on 23,667 SNPs. (c) PCA plot showing population differentiation of populations only in the Guanapo drainage (CA, GP, TU, TY) based on 23,667 SNPs

In October 2015, we collected 65 adult male Trinidadian guppies (*Poecilia reticulata* Peters) using butterfly nets from each of six locations on the south slope of the Northern Range mountains in Trinidad. The comparison we were most interested in was between populations that experienced recent human‐mediated gene flow (Caigual‐CA and Taylor‐TY) and populations in similar environments that did not have a recent history of gene flow (Naranjo‐NJ, Quare‐QU, and Tumbassun‐TU). These five populations were all from low‐order headwater streams with highly similar abiotic and biotic properties (Figure 2a; Table 1). These are considered “low‐predation” sites because they are located above waterfalls and harbor simple fish communities that lack the major piscivorous predators such as *Crenicichla alta * found in lowland rivers. These upstream sites are also characterized by low‐resource availability due to thick forest canopy cover and high guppy densities. In general, abiotic and biotic variation among headwater sites is much less than variation between headwater and mainstem environments (Grether et al., 2001; Magurran, 2005). From these five headwater sites, we collected individuals at the upstream‐most extent of where guppies could be accessed in order to target the populations that should be experiencing highest genetic drift and harbor lowest levels of within‐stream genetic variation (Baillie, 2012). The sixth site where guppies were collected (Guanapo‐GP) is a mainstem locality from the same drainage as CA, TY, and TU. This lowland site is characterized by higher water temperatures, high resources, and high levels of predation (Gilliam, Fraser, & Alkins‐Koo, 1993; Torres Dowdall et al., 2012). We included the GP site because 150 guppies originating from this site were introduced upstream of CA and TY in 2009 (see Travis et al., 2014), leading to ~20 generations of gene flow and admixture between GP × TY and GP × CA populations (Fitzpatrick et al., 2016, 2015).

**Table 1:** Sample collection data

Stream	Collection site coordinates	Habitat type	Predation level	Avg. stream temperature (°C)	Recent gene flow?	No. fish genotyped
Guanapo (GP)	N10°38.402′ W61**°**14.896′	Mainstem	High	30.1	Source	15
Caigual (CA)	N10**°**42.768′ W61**°**16.289′	Headwater	Low	24.7	Gene flow	17
Taylor (TY)	N10**°**42.472′ W61**°**16.277′	Headwater	Low	24.6	Gene flow	18
Naranjo (NJ)	N10**°**41.143′ W61**°**13.948′	Headwater	Low	24.9	None	14
Quare (QU)	N10**°**40.559′ W61**°**11.802′	Headwater	Low	24.9	None	14
Tumbassun (TU)	N10**°**42.370′ W61**°**15.728′	Headwater	Low	24.4	None	13

Note

Stream name and ID correspond to Figure 2a, habitat type is based on first‐order (headwater) or fourth‐order (mainstem) streams, predation level is based on presence (high) or absence (low) of a diverse piscivorous fish community, number of fish genotyped refers to the number per site included in the RADseq analyses that passed filtering criteria.

In future work, it will be important to evaluate acute stress response of both sexes, especially given that sex‐specific responses to stressors such as temperature are common (Baer & Travis, 2000). However, in this study we collected only mature males to avoid confounding effects of female pregnancy in assessing stress responses. Male maturity was confirmed under a microscope by determining that the apical hood was even with or beyond the tip of the gonopodium. Fish were transported to the laboratory in Nalgene^®^ (Rochester, NY, USA) bottles filled with local stream water and held in individual, aerated tanks. All individuals were anesthetized in dilute MS‐222, weighed, and photographed on the day of capture to estimate initial body condition (weight divided by standard length). Photographs were taken of fish spread laterally on a white background alongside a metric ruler using a Canon EOS Rebel XSi SLR digital camera (Canon U.S.A., Inc., Melville, NY, USA).

### 2.2 Stress exposure experiments

All experimental procedures took place at Jogi Ramal's tropical field station in Verdant Vale, Trinidad. We randomly assigned each of the 65 male guppies per population to one of three acute stress exposure experiments: (a) maximum temperature tolerance (30 guppies/population), (b) starvation (20 guppies/population), or (c) toxicity (15 guppies/population). All fish were held in constant laboratory environment conditions for two nights and one full day before experiments began.

Maximum temperature tolerance in guppy populations was determined by estimating critical thermal maximum (CTM) for 30 guppies per population. Guppies were starved for one day prior to the CTM experiments to avoid energy expenditure for digestion during the experiment. We used an insulated rectangular (34.5 × 21.1 × 56.6 cm) Coleman 25‐quart cooler filled with a constant volume (7.6 L) of water that was circulated with a 2.8 watt Rio Plus 90 Aqua pump and aerated with two air stones. We tested six guppies per CTM assay, randomly placing each individual into a mesh container that was secured to the bottom of the cooler. After a 5‐min adjustment period, water was heated at a constant rate of 0.4°C/min with two immersion coils connected to a PHC Enterprise voltage transformer set to 75 V. Temperature was recorded with a handheld YSI^®^ Pro20 Multiparameter Meter (YSI Incorporated, Yellow Springs, OH). CTM was recorded as the temperature at which an individual showed initial loss of equilibrium. Usually, this point was preceded by several muscle spasms. As soon as a guppy reached CTM, it was removed with a plastic spoon and placed into a recovery tank. Three fish did not recover from the CTM experiment and were not included in analyses.

To assess population differences in the ability to withstand low‐resource environments, we starved individually housed guppies for 120 hr. We based this interval on a previous study that used a similar duration for its guppy starvation treatment (Bowes, Lafferty, & Thorp, 2014) as well as the observation that in the wet season headwater streams tend to have high stream‐flow events every 5 days on average (Kohler et al., 2012). We calculated body condition at the start of the experiment (0 hr) and again after 120 hr without any food. To determine whether starvation over this duration would produce any measurable effects on body condition, we included a set of five randomly assigned control guppies per population that were held in exactly the same conditions with the exception of being fed ground Tetramin^®^ tropical fish flakes (Spectrum Brands, Inc., Cincinnati, OH) ad lib once a day at 18:00. We measured body condition of control fish before and after 120 hr. To standardize the effects of starvation across individuals and populations, we quantified a starvation index for each individual, defined as body condition at 0 hr minus body condition at 120 hr. A positive starvation index at the end of the experiment indicated a reduction in body condition, whereas negative values indicate the fish gained weight. Thus, we interpreted higher values of starvation index as corresponding to worsened ability to cope with a lack of resources. We used a Welch two sample *t* test to determine whether fish that were starved had overall higher starvation indices than control fish that were fed during the experiment. Although groups had unequal sample sizes, assumptions of equal variance and normality were met.

Finally, we evaluated resistance to exposure to toxicity by subjecting group tanks to a toxic level of copper sulfate (CuSO_4_). Anthropogenic sources of copper pollution appear in aquatic systems from mine washing, agricultural leaching, and direct application as algaecide and molluscicide (Helfman, 2007). Given the remote location of the headwater guppy sites in our study, it is unlikely the focal populations had any previous exposure to the metal toxin. In this experiment, 15 males per population were held in group tanks separated by population with 4 L of water treated with slightly less than the previously established LC_50_ level of CuSO_4_ for guppies (0.05 mg/L compared to 1.17 mg/L established by Park & Heo, 2009). Tanks were aerated and also treated with STRESS COAT^®^ (API, Mars Fishcare) and AmQuel PLUS^®^ (Kordon, Oakland, CA). Guppies in the toxicity experiment were fed ground fish flakes ad lib once a day at 18:00 p.m. Tanks were checked every 12 hr and dead fish were removed. We assessed the survival rate for each tank after 96 hr.

### 2.3 RADseq library preparation

Caudal peduncle tissue from 30 individuals per population was collected from euthanized fish from the thermal tolerance experiment and preserved in 95% ethanol. Genomic DNA was purified from muscle tissue using Qiagen^TM^ DNeasy Blood & Tissue extraction kits and quantified using the Qubit dsDNA HS Assay kit (Thermo Fisher Scientific). A single RAD library was prepared with 96 individuals (Table 1) following the bestRAD protocol (Ali et al., 2016). Briefly, we normalized DNA from every sample to a final concentration of 150 ng in a 10 μl volume, digested DNA using the SbfI restriction enzyme (New England Biolabs, NEB), and ligated digested samples to individually barcoded adapters. Samples were pooled and sheared to an average length of 500 bp on a Covaris sonicator. We used the Illumina NEBNext Ultra DNA Library Prep Kit to repair blunt ends and ligate NEBNext Adaptors onto DNA fragments. Agencourt^®^ AMPure XP beads (Beckman Coulter) were used to size select from the DNA fragments with an average of 600 bp. The final library was submitted to the RTSF Genomics Core at Michigan State University and sequenced in one lane with paired‐end 150‐bp reads on an Illumina HiSeq 4000.

### 2.4 Acute stress analyses

We used linear mixed models to analyze the effects of population differences on CTM and starvation index. For modeling variation in CTM, we used stream as the fixed factor. Initial weight was included as a random effect as were hierarchically nested factors of CTM run and container position. For modeling variation in starvation index, we used stream as the fixed factor and included initial weight as a random effect. Both CTM and starvation index were modeled using maximum likelihood, and significance of the population effect was tested using likelihood ratio tests against the null model that included only random effects. Residual plots were used to determine whether model assumptions of normality and homoscedasticity were met. All models were carried out with package “lme4” in R (Bates & Maechler, 2009). To evaluate the effects of gene flow on resistance to the novel copper sulfate stress, we used interval‐censored survival methods (Finkelstein, 1986). We coded each individual tested in the copper sulfate trials as either perishing at an unknown time between two tank checks or surviving to the end of the trial (right‐censored). We fit survival curves and tested for differences in survival distributions among groups using a chi‐square permutation test based on logrank scores in the R package “interval” (Fay & Shaw, 2010). We conducted two analyses using two different grouping schemes, one which considered each of the six populations as a separate group (to identify whether individual populations differed significantly in survival after copper sulfate exposure) and another which split individuals into three groups by origin in either the mainstem source population, headwater populations with a recent history of gene flow, or headwater populations with no gene flow (to test whether gene flow treatment affected survival).

### 2.5 RADseq analyses

We used Stacks (Catchen, Hohenlohe, Bassham, Amores, & Cresko, 2013) to demultiplex, filter, and trim adapters from the sequencing data with the process_radtags function. We required a perfect barcode and partial restriction site match. We removed duplicate read pairs using the clone_filter function in Stacks. Forward‐read sequences were aligned to the Trinidadian guppy genome assembly (Künstner et al., 2016) using GSnap (Wu & Watanabe, 2005). We required unique alignments, allowing for a maximum of five mismatches, the presence of up to two indels, and no terminal alignments. Reference‐aligned reads were analyzed using the ref_map function in Stacks to identify single nucleotide polymorphisms (SNPs) and call genotypes. We kept only SNPs that were present in all six populations and genotyped in at least 60% of the individuals. We also removed several individuals which had fewer than 1,000 loci in the final dataset and removed loci with a minor allele frequency <0.05 using VCFtools v0.1.12a (Danecek et al., 2011). In total, we identified 23,667 SNPs that met our filtering criteria.

### 2.6 Genome‐wide genetic diversity and divergence

We used the R package PopGenome v2.2.4 (Pfeifer, Wittelsbürger, Ramos‐Onsins, & Lercher, 2014) to characterize genome‐wide nucleotide diversity and pairwise genetic divergence (*F*_ST_) between the source population (GP) and the other populations (including those with and without a history of gene flow). We calculated both statistics over adjacent 50‐snp windows and plotted diversity and pairwise *F*_ST_ using local regression (LOESS; Cleveland, 1979) over each chromosome. We conducted a principal component analysis (PCA) on the genomic data using the R package adegenet v.2.1.1 (Jombart, 2008). We performed one PCA analysis using all populations and another PCA analysis using only populations in the Guanapo drainage (CA, GP, TU, TY) to detect fine‐scale differences within this drainage. We visualized PCA results using the first two principal components as *X* and *Y* coordinates.

### 2.7 Inbreeding

We used multiple methods to characterize inbreeding for the study populations. We calculated genome‐wide inbreeding coefficients (*F*) using VCFtools for each individual. We characterized variation in inbreeding among individuals using *g*2, a metric of correlation of heterozygosity across markers (David, Pujol, Viard, Castella, & Goudet, 2007; Miller & Coltman, 2014), for each population as well as for all populations combined using the inbreedR package (Stoffel et al., 2016). Finally, we used PLINK v1.07 (Purcell et al., 2007) to identify long runs of homozygosity (LROH) for each individual. We used sliding windows of 100 SNPs and we set the minimum size of LROH to 1 Mb, with a maximum gap of 500 kb, 1 heterozygote allowed per LROH, and 10 missing loci allowed per LROH.

### 2.8 Outliers

To find loci potentially associated with maintenance of local adaptation in headwater populations with a history of gene flow from the mainstem source, we identified outlier loci exhibiting higher divergence than expected using BayeScan v2.1 (Foll & Gaggiotti, 2008). We used default settings for the MCMC chain (20 pilot runs, first 50,000 runs discarded as burn‐in, thinning interval of 10, and 5,000 outputted iterations) and a prior odds of 10 for the neutral model. We conducted one run using allele frequencies for all populations with a known history of gene flow from the mainstem source (GP, CA, and TY) as well as one run with all populations in the same drainage as GP (GP, CA, TY, and TU). We used a false discovery rate of 0.05 to identify the loci with the best evidence for association with adaptive divergence between the headwater and mainstem populations. For each SNP locus identified as an outlier, we examined allele frequencies in both the mainstem population and the headwater populations in the same drainage to identify patterns of genetic variability potentially associated with local adaptation to headwater environments. Finally, we used the NCBI Genome Data Viewer for the published *Poecilia reticulata* genome to identify the position of each SNP relative to nearby genes in order to identify potential functional importance of these outliers.

## 3 RESULTS

### 3.1 Stress exposure

**Figure 3:**
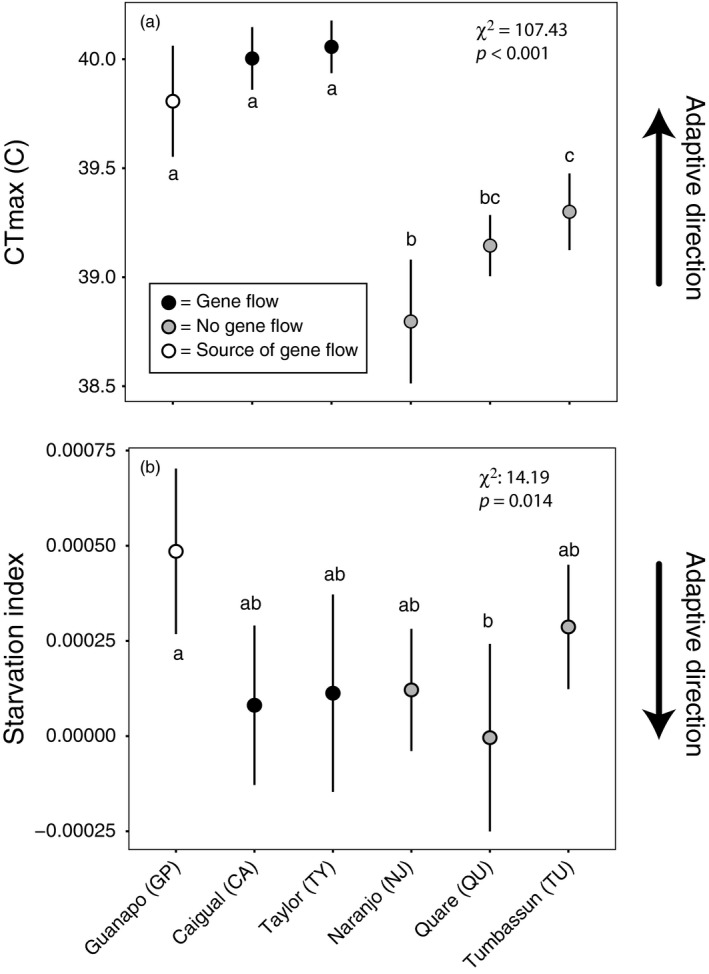
Population responses following acute stress exposure to (a) critical thermal maxima, and (b) starvation for 120 hr. Arrows on the right of plots indicate predicted adaptive direction in headwater environments. Means and 95% confidence intervals are shown and chi‐squared statistics correspond to the likelihood ratio test described in the text where lowercase letters indicate significant differences among populations based on post hoc Tukey's HSD tests

We found that guppy populations differed in their responses to acute stressors and with respect to each stress type. Despite cooler water temperatures in all headwater environments (Table 1), the two sites that previously received gene flow showed highest thermal tolerances and were significantly different from other headwater populations, but not different from the Guanapo (GP) mainstem site that provided the source of gene flow (Figure 3a). Thermal tolerance of Naranjo (NJ) fish was significantly lower than it was for Tumbassun (TU) fish, while this tolerance was intermediate for Quare (QU) fish.

We confirmed that guppies that experienced starvation had higher overall starvation indices than those that were fed in control treatments, *t*(40) = −8.61, *p* < 0.0001. All headwater populations started with higher body condition compared to Guanapo (GP) guppies from the lowland mainstem site; however, Tumbassun (TU) guppies had significantly higher body condition than all other headwater populations (Supporting Information Figure S1). Headwater populations tended to withstand starvation stress better than the mainstem Guanapo population, although the only significant difference was between Guanapo and Quare (Figure 3b).

**Figure 4:**
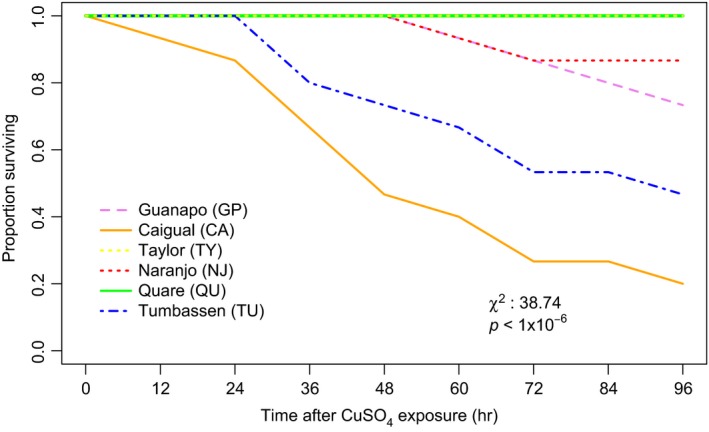
Survival rates for guppy populations exposed to copper sulfate (CuSO_4_) at the LC_50_/96 hr level

Finally, we documented substantial variation among populations with respect to survival rate when exposed to copper sulfate (Figure 4). The two populations that previously received gene flow highlighted two extremes with only 20% survival rate in Caigual compared to 100% survival rate in Taylor after 96 hr exposure. One other population (Quare) had 100% survival and the three remaining populations were intermediate, ranging from 50% to 80% survival. Survival distributions differed significantly among populations (Figure 4; *χ* ^2^ = 38.74, *p* < 1 × 10^−6^) but not among the source, gene flow, and no gene flow groups (Supporting Information Figure S2; *χ* ^2^ = 3.32, *p* = 0.1906).

### 3.2 Genome‐wide genetic divergence and diversity

**Figure 5:**
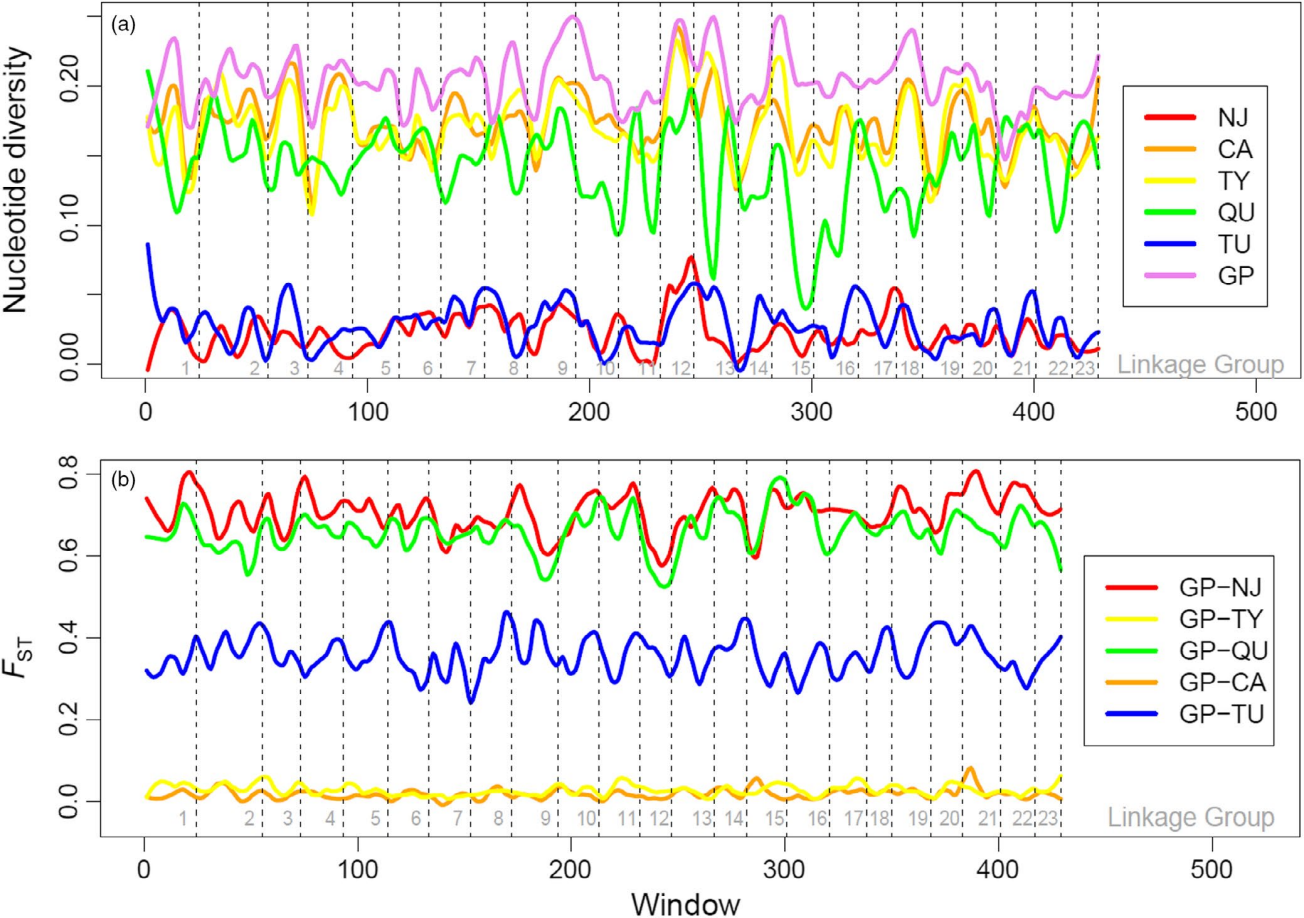
(a) Genome‐wide genetic diversity for each study population. Lines show fitted local regression of per‐SNP nucleotide diversity calculated over adjacent 50‐SNP windows. (b) Genome‐wide genetic divergence between the mainstem source population and each headwater population. Lines show fitted local regression of *F*_ST_ calculated over adjacent 50‐SNP windows. Dotted lines delineate linkage groups

Per‐SNP nucleotide diversity was highest for the Guanapo (GP) mainstem source site (mean = 0.20, range 0.08–0.31) and lowest for headwater populations Tumbassun (TU mean = 0.03, range 0–0.20) and Naranjo (NJ mean = 0.02, range 0–0.23) with no recent history of gene flow. Headwater populations that experienced recent gene flow from the GP source exhibited diversity similar to that of the source (CA mean = 0.17, range 0.05–0.30; TY mean = 0.17, range 0.04–0.30), and headwater population QU also exhibited intermediate diversity (mean = 0.14, range 0–0.28) despite no known recent history of gene flow (Figure 5a).

For the PCA analysis using all individuals, the first two components explained 37.6% and 25.3% of the variability in the data, with all other components explaining <5% of the variability. Based on these first two components, individuals from populations NJ and QU were highly distinct from both each other and from individuals in the Guanapo drainage (Figure 2b). For the PCA analysis restricted to the Guanapo drainage, the first two components explained less variability (11% and 4% of variability). The first axis differentiated the no gene flow population (TU) from the source population and from the populations that previously received gene flow from the source, with remaining axes mostly explaining variability in the source population. Individuals from CA and TY were clustered closely together, while individuals from the GP source population exhibited more variability (Figure 2c).

*F*_ST_ was highest between GP and headwater populations in other drainages (GP‐NJ *F*_ST_ mean = 0.71, range 0.29–0.91; GP‐QU *F*_ST_ mean = 0.66, range 0.31–0.90), intermediate between GP and the headwater population in the same drainage with no gene flow (GP‐TU *F*_ST_ mean = 0.36, range 0.001–0.74), and low between GP and the populations with a history of gene flow from GP (GP‐CA mean *F*_ST_ = 0.02, range −0.06 to 0.18; GP‐TY mean *F*_ST_ = 0.03, range −0.08 to 0.16; Figure 5b).

### 3.3 Inbreeding

**Figure 6:**
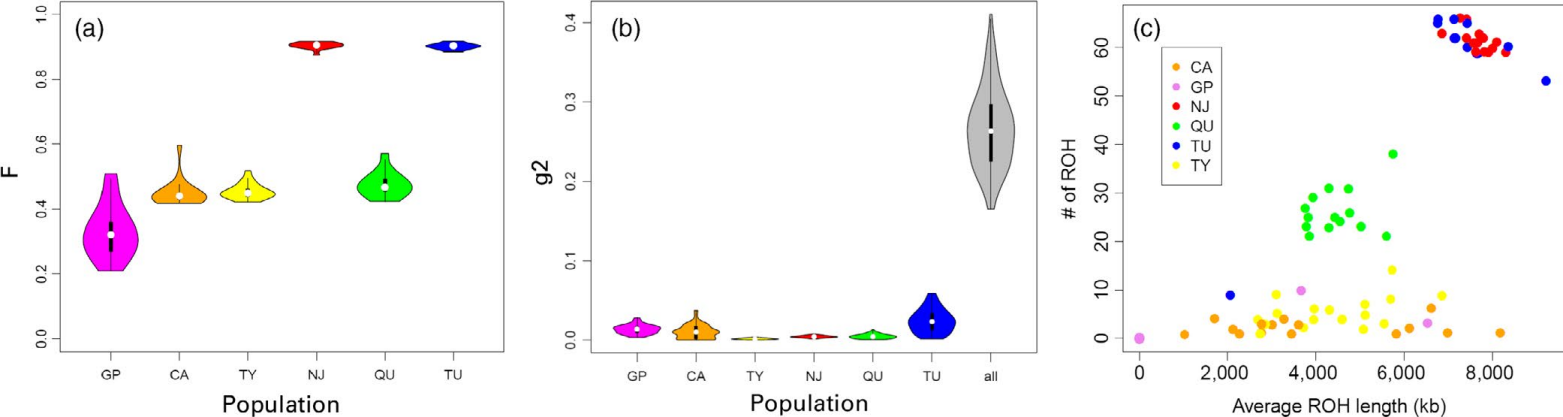
(a) Violin plot showing distributions of individual inbreeding values for each population where larger values indicate a higher frequency of inbreeding. (b) Violin plot of bootstrapped values of the *g*2 statistic for each population where larger values indicate increased variation in inbreeding among individuals. (c) Autozygosity statistics. The number of runs of homozygosity detected for each individual is plotted on the *Y*‐axis, and the average length of these runs of homozygosity is plotted on the *X*‐axis

Individual inbreeding estimates were lowest for GP (mean *F* = 0.33, range 0.21–0.51) and highest for the two headwater populations that exhibited lowest nucleotide diversity (NJ mean *F* = 0.90, range 0.88–0.92; TU mean *F* = 0.90, range 0.89 to 0.92; Figure 6a). Populations that received gene flow from the mainstem GP population exhibited individual inbreeding near that of the source (CA mean *F* = 0.45, range 0.42–0.60; TY mean *F* = 0.46, range 0.42–0.52). Variation in inbreeding was high across populations (*g*2 = 0.271) and generally near zero within populations (Figure 6b). Individuals in headwater populations with high average *F* values tended to exhibit many long runs of homozygosity (LROH) (>50) with high average LROH length (>6 Mb; Figure 6c). In contrast, most individuals from the mainstem GP population exhibited no detectable LROH. Populations with a recent history of gene flow tended to exhibit few LROH, although the length of LROH varied considerably. LROH for individuals in population QU were intermediate in terms both number and length. The headwater population with higher genetic diversity also exhibited levels of individual inbreeding comparable to levels seen in populations experiencing gene flow (QU mean *F* = 0.48, range = 0.42–0.57).

### 3.4 Outliers

Both BayeScan analyses identified the same set of five loci as outliers with a false discovery rate of 0.05. All of these loci were variable in the mainstem GP population but fixed in headwater populations. Three loci were distributed throughout the genome (linkage groups 1, 5, and 8), while two were in close association with one another (<100 kb apart) on linkage group 17. Three of the five loci were found within intronic regions of protein‐coding genes (Supporting Information Table S1), with functions in skin pigmentation (gene *bnc1*, linkage group 1), endoplasmic reticulum function (gene *emc10*, linkage group 8), and cyclic AMP production/signaling cascades (gene *adcy8*, linkage group 17).

## 4 DISCUSSION

Small populations with high genetic load may be constrained in their ability to withstand abrupt environmental stress. Gene flow may ameliorate these genetic constraints by reducing inbreeding depression and introducing adaptive alleles, or could decrease fitness by introducing maladaptive alleles. We found higher genomic variation and less inbreeding in headwater populations with a history of recent gene flow compared to populations without recent gene flow. Populations with gene flow either outperformed or performed similarly to "no‐gene flow" analogues when exposed to familiar stressors, but did not show a consistent response to a novel stress. We discuss these results in light of the following generalizations: (a) inbreeding depression is often worse under stressful conditions (Armbruster & Reed, 2005); (b) gene flow can relieve genetic load but may introduce maladaptive alleles (Slatkin, 1985); and (c) many natural populations are in decline, increasingly isolated, and subjected to new and more extreme stress (Frankham et al., 2017). The extent to which reduced genetic variation imposes physiological constraints to stress tolerance, and how gene flow relieves those constraints is not currently known.

Local adaptation and inbreeding may create interactive effects that compound maladaptation to certain stresses. For example, the ability to tolerate extreme temperatures is mechanistically tied to upregulation of heat shock proteins (HSPs), and populations experiencing different levels of heat stress in their natural environment exhibit differences in their ability to respond to heat stress through upregulation of HSPs (Feder & Hofmann, 1999; in fish, Narum & Campbell, 2015). Evidence for heritable genetic variation in HSP expression (marine turtles; Tedeschi et al., 2016) and local adaptation to heat stress (in corals; Bay & Palumbi, 2014; Dixon et al., 2015) suggests that selective forces can tune the transcriptional heat shock response according to local thermal conditions. However, there is also evidence that inbreeding alone can interfere with the expression of HSPs and heat shock responses (in insects; Pedersen, Kristensen, & Loeschcke, 2005; Franke & Fischer, 2015), suggesting that local adaptation may be insufficient to deal with heat stress in small populations. A broad knowledge of population history (in terms of past inbreeding, gene flow, and local conditions) may thus be useful in understanding and predicting how increasing temperatures affect small populations.

In this study, headwater populations with no history of gene flow were all maladapted to heat stress in comparison with the mainstem population from a warmer environment. While genomic data indicated that populations without a history of gene flow varied in their degree of inbreeding and level of genetic diversity, the degree of maladaptation in these populations did not seem to depend strongly on the degree of inbreeding, as two highly inbred populations (NJ and TU) exhibited critical thermal maximums (CTMs) similar to a more outbred population (QU). As such, maladaptation to heat stress in this case seems to be tied more to local selective forces than random drift. However, headwater populations that had experienced recent gene flow exhibited CTMs similar to the source population, indicating that gene flow alleviated maladaptation to heat stress in this case, fitting the predicted scenario in Figure 1b. It is important to note here that the recipient and source populations originated from the same drainage, and as such we cannot state with certainty whether gene flow per se from any population would alleviate heat stress response or whether the specific alleles contributed from the source used in this case were responsible. Further investigation into the specific mechanism by which gene flow alleviates maladaptation to heat stress would be useful for better understanding how gene flow affects stress responses. Specifically, understanding transcriptional responses to stress in populations with varying histories of gene flow and inbreeding would be useful. Previous work has shown that local adaptation to heat stress can be highly polygenic (Bay & Palumbi, 2014), which would suggest that exchange of alleles across the whole genome rather than in specific regions would be particularly helpful in alleviating maladaptation to heat stress.

While this result shows that gene flow can alleviate maladaptation to some stresses, particularly stresses that are familiar to the source population, theory predicts that gene flow should aggravate maladaptation to stresses that are unfamiliar to the source population by homogenizing allele frequencies among populations and thus disrupting locally adapted differences in allele frequency in the recipient population (Lenormand, 2002; Rasanen & Hendry, 2008). We expected to see this effect with regard to a stress familiar to low‐resource adapted headwater populations (starvation). However, in this case gene flow did not seem to aggravate maladaptation, as the mainstem population (adapted to a high resource environment) was maladapted to starvation relative to all headwater populations, regardless of gene flow history. This result has two possible explanations that we cannot distinguish in our study: (a) headwater × mainstem hybrids are more plastic in starvation response than pure mainstem genotypes; or (2) strong selective forces maintained differences in allele frequencies in headwater populations at loci that confer adaptation to low‐resource levels. The existence of *F*_ST_ outliers provides support for the latter explanation, although increased plasticity may also contribute. Constant high gene flow between heterogeneous environments is expected to increase plasticity (Crispo, 2008; Lind, Ingvarsson, Johansson, Hall, & Johansson, 2011; Sultan & Spencer, 2002), but interactions between new gene flow, inbreeding, and plastic stress response are poorly understood. Experimental studies that manipulate gene flow and inbreeding and test short‐term stress response and long‐term adaptive potential would improve understanding of these complex interactions.

The existence of outlier alleles that remained fixed in headwater populations despite gene flow from the diverse source population suggests possible maintenance of some locally adaptive alleles. The repeated and independent evolution of the classic low‐predation guppy phenotype (i.e., slow life history and large body size) in headwater environments is thought to be largely a result of density‐dependent selection driven by low‐resource availability and high population densities (Travis et al., 2014). In this study, we are unable to determine whether outlier‐identified alleles underlie increased tolerance to starvation. Nonetheless, it is likely that strong selection in headwater environments maintains genetic adaptations to low resources and high population densities. In *Drosophila*, starvation results in a large number of transcriptional changes throughout the genome, and starvation‐resistant populations show different patterns of regulation than nonresistant populations (Harbison, Yamamoto, Fanara, Norga, & Mackay, 2004). These differences in regulation, however, appear to be related to a small number of quantitative trait loci (~12), potentially involving genes that have broad regulatory effects (Harbison, Chang, Kamdar, & Mackay, 2005). Selection acting on starvation‐related regulatory loci is therefore a possible mechanism for the maintenance of local adaptation in headwater populations of guppies. Studies quantifying transcriptional changes in guppies under starvation as well as fine mapping of loci responsible for variability in starvation resistance would be a means of evaluating this mechanism.

Reactions to novel stressors in small populations are related to a population's history of inbreeding as well as the variability of the environment during inbreeding. For example, inbred populations that have experienced more variable environments tend to be better able to cope with novel stress (Reed, Lowe, Briscoe, & Frankham, 2003). Contrary to the hypothesis that variation provided by gene flow would confer higher tolerance to a novel stress, in our study populations the degree of maladaptation to a novel stress (copper) seemed to follow a pattern unrelated to genetic diversity or divergence among populations. High resistance to copper exposure in TY, a population inhabiting an especially flood‐prone and resource‐poor headwater stream environment (Fitzpatrick, Torres Dowdall, Reznick, Ghalambor, & Funk, 2014), supported a relationship between environmental variability and response to novel stress. However, the extreme difference in response between TY and CA, both of which experienced homogenizing gene flow from the same source, is difficult to explain. Resistance to novel stress can show strong lineage effects (Reed, Briscoe, & Frankham, 2002), suggesting that founder effects could be involved in determining response to novel stressors in isolated headwater populations. Copper ions are highly toxic to fish; accumulation of copper in gill, kidney, and liver tissues has been shown to affect tissue and cellular morphology as well as physiological function in several species (Benoit, 1975; De Boeck et al., 2001; Brungs, Leonard, & McKim, 1973). Determining the histological and genetic mechanisms that conferred survival to acute copper poisoning in these populations would be informative for understanding how fish resist heavy metals. Studies conducted in fish as well as in invertebrates have independently identified several genes associated with resistance to heavy metals, including copper, is associated with changes in the expression of several genes, including metallothioneins which bind metal ions (Hamilton & Mehrle, 1986; Roesijadi, 1992) and transport proteins such as ATP7a (Camakaris et al., 1995) and ABC transporters (Broeks, Gerrard, Allikmets, Dean, & Plasterk, 1996; Long, Li, & Cui, 2010) that move metal ions out of the cell, making these ideal candidates for genes conferring resistance to copper stress and potential targets for future study.

Our case study adds to the generalization that effects of gene flow are trait specific, but also suggests that some trait responses (such as thermal tolerance and starvation) may be predictable based on previous exposure of source and recipient populations. The ability to cope with increased and novel stresses will be extremely important in determining the fate of small populations in a changing world (Hoffmann & Sgro, 2011). Determining how the loss of genetic variation in small populations affects short‐term stress response and long‐term adaptive potential to environmental change is an urgent concern (Frankham et al., 2017). Other studies have identified a tension between conservation interventions designed to bolster populations in the short‐term and those designed to increase long‐term adaptive capacity (Derry et al., 2019). Genetic rescue is often included in the former category; however, this study shows that genetic rescue may also improve individual's long‐term capacity to respond to novel environmental stress. We would emphasize that the ability of individuals to endure chronic or novel stress is a component of fitness, but that the relationship between stress responses and overall fitness likely depends on a number of other factors, including the realized reproductive success of individuals exposed to stress, potential trade‐offs between the ability to respond to stress and other fitness components, and the frequency of exposure to stress. As demonstration of positive fitness outcomes of restored gene flow accumulates (Hasselgren et al., 2018; Hogg, Forbes, Steele, & Luikart, 2006; Hufbauer et al., 2015; Johnson et al., 2010; Kronenberger et al., 2018; Madsen, Shine, Olsson, & Wittzell, 1999; Robinson et al., 2017) and calls for gene flow manipulation grow louder (Ralls et al., 2018; Whiteley et al., 2015), it will also be critical to understand the extent and mechanisms by which new gene flow affects immediate and long‐term response to environmental stress. In some cases of abrupt stress, such as high‐temperature exposure in our study, gene flow may increase initial tolerance and reduce extinction probability. Other scenarios, such as metal pollution in our study, may be less generalizable. The genomic architecture underlying maladaptive and adaptive stress responses and eventual adaptive evolution to a range of environmental stressors is likely to be identified over the next few years. Combined with demographic analyses in experimental and natural populations, these studies will provide new insights and better mechanistic understanding into interactions between contemporary evolution and population persistence under increasingly harsh environments.

## Data Availability

Data for this study are available at https://doi.org/10.5061/dryad.rf79641.
