# Supplementary material for: Does gene flow aggravate or alleviate maladaptation to environmental stress in small populations?
Source: Evol Appl. 2019 Feb 4;12(7):1402–16. doi: 10.1111/eva.12768 (PMC6691220; doi:10.1111/eva.12768)
Supplement: Supplementary file 1 [file EVA-12-1402-s001.docx]

**Supplemental figure S1**


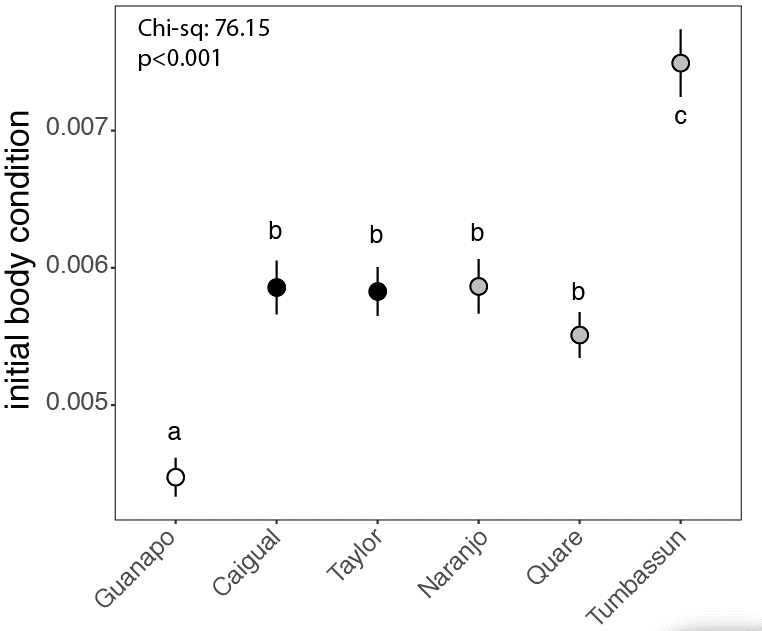


Initial body condition (weight divided by length) of male guppies on day of sampling for the mainstem source of gene flow (open circle), two headwater populations with gene flow history (black circle), and three headwater populations with no gene flow history (grey circle). Population means and 95% confidence intervals are shown. Chi-squared statistics correspond to the likelihood ratio test described in the text and lowercase letters indicate significant differences among populations based on *post hoc* Tukey’s HSD tests.
