# Supplementary figures and images for: Does gene flow aggravate or alleviate maladaptation to environmental stress in small populations?
Source: Evol Appl. 2019 Feb 4;12(7):1402–16. doi: 10.1111/eva.12768 (PMC6691220; doi:10.1111/eva.12768)

Proportion surviving

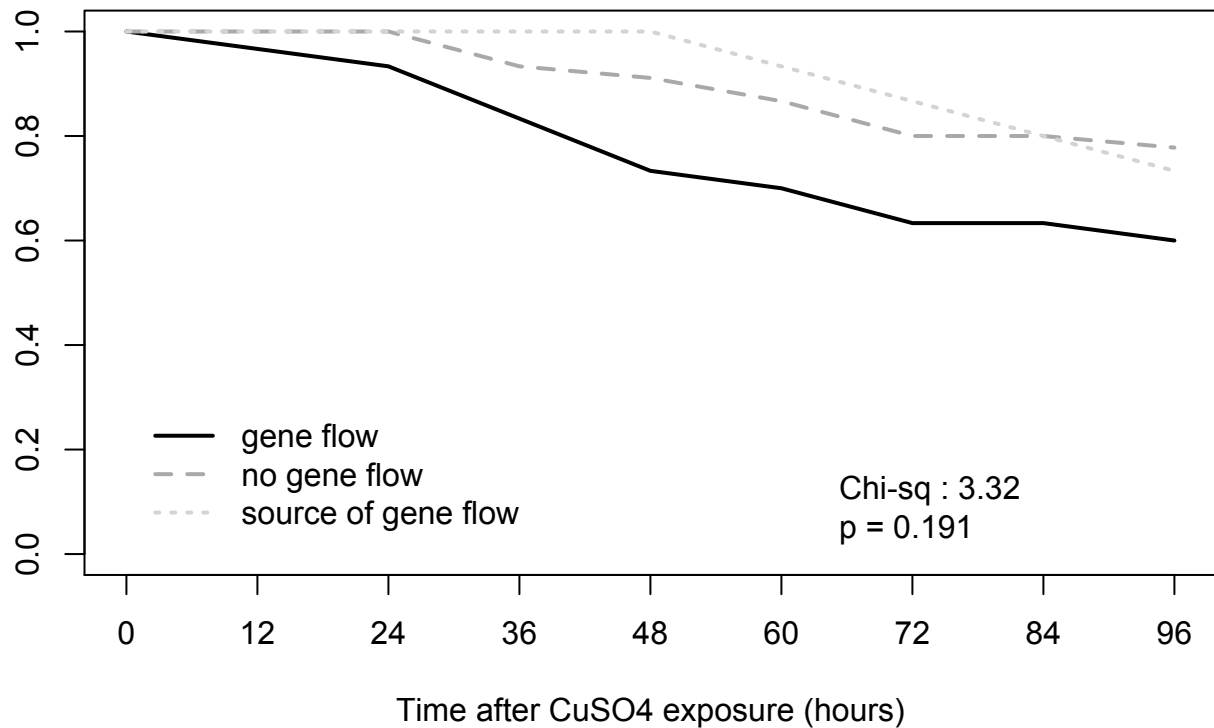

Supplement: Supplementary file 2 [file EVA-12-1402-s002.pdf]
